# A New Chytridiomycete Fungus Intermixed with Crustacean Resting Eggs in a 407-Million-Year-Old Continental Freshwater Environment

**DOI:** 10.1371/journal.pone.0167301

**Published:** 2016-12-14

**Authors:** Christine Strullu-Derrien, Tomasz Goral, Joyce E. Longcore, Jørgen Olesen, Paul Kenrick, Gregory D. Edgecombe

**Affiliations:** 1 Department of Earth Sciences, The Natural History Museum, Cromwell Road, London, United Kingdom; 2 Imaging and Analysis Centre, The Natural History Museum, Cromwell Road, London, United Kingdom; 3 School of Biology and Ecology, University of Maine, 5722 Deering Hall, Orono, ME, United States of America; 4 Natural History Museum, University of Copenhagen, Universitetsparken, Denmark; University of Wisconsin Madison, UNITED STATES

## Abstract

The 407-million-year-old Rhynie Chert (Scotland) contains the most intact fossilised remains of an early land-based ecosystem including plants, arthropods, fungi and other microorganisms. Although most studies have focused on the terrestrial component, fossilised freshwater environments provide critical insights into fungal-algal interactions and the earliest continental branchiopod crustaceans. Here we report interactions between an enigmatic organism and an exquisitely preserved fungus. The fungal reproductive structures are intermixed with exceptionally well-preserved globular spiny structures interpreted as branchiopod resting eggs. Confocal laser scanning microscopy enabled us to reconstruct the fungus and its possible mode of nutrition, the affinity of the resting eggs, and their spatial associations. The new fungus (*Cultoraquaticus trewini* gen. et sp. nov) is attributed to Chytridiomycota based on its size, consistent formation of papillae, and the presence of an internal rhizoidal system. It is the most pristine fossil Chytridiomycota known, especially in terms of rhizoidal development and closely resembles living species in the Rhizophydiales. The spiny resting eggs are attributed to the crustacean *Lepidocaris rhyniensis*, dating branchiopod adaptation to life in ephemeral pools to the Early Devonian. The new fungal interaction suggests that, as in modern freshwater environments, chytrids were important to the mobilisation of nutrients in early aquatic foodwebs.

## Introduction

Direct fossil evidence of fungal interactions and fungal diversity in general is relatively limited, principally because investigation of the fossil record is underdeveloped [1]. Paradoxically, one of the few well-studied fossil fungi sites is also one of the oldest. The 407-Myr-old Rhynie Chert (Scotland, UK) preserves its biotic components in exquisite detail [2]. The cherts formed from erupted hydrothermal fluids that periodically inundated vegetation on a low-energy alluvial plain formed by a braided river channel. Minor variations in topology across the floodplain gave rise to habitats that ranged from terrestrial to fully freshwater [3]. Fungal interactions in the terrestrial environments include the earliest evidence of plant parasitism and symbiosis (e.g. [4–8]), whilst interactions in freshwater involve fungi and algae [9, 10]; the latter also contain the earliest continental branchiopod crustaceans [11–13]. Fossil remains of Chytridiomycota (chytrids) and Blastocladiomycota (blastoclads) have been reported from both environments [8–10, 14–20]. These true Fungi are early diverging lineages in the fungal tree of life that reproduce with motile spores (zoospores). The body or thallus is formed by organs of reproduction (zoosporangia and resting sporangia) arising from a vegetative part consisting of rhizoids. The most prominent morphological feature of the thallus is the zoosporangium, a sac-like structure bearing one or more discharge tubes or exit papillae. Zoosporangia are thin-walled whereas resting sporangia are thick-walled structures that may germinate to produce a sporangium after a dormant period. Fossil remains of chytrids (Chytridiomycota and Blastocladiomycota) have been reported from both environments [8–10, 14–20]. Chytrids are true Fungi that are an early diverging lineage in the fungal tree of life [21, 22]. Today, chytrids and blastoclads are ubiquitous, occurring in diverse habitats from the tropics to the arctic regions [23]. They develop in terrestrial habitats [24] as saprotrophs or obligate parasites of plants [22]. They are the dominant parasites of algae and plankton in aquatic ecosystems, and recently they have been recognised as playing a fundamental role in zooplankton production and aquatic foodwebs [25]. Here we document a new freshwater chytrid from the Rhynie Chert interacting with structures of unknown affinity. Intermixed with the fungal reproductive structures are spinose fossils that we identify as resting eggs of the branchiopod crustacean *Lepidocaris rhyniensis*, the post-embryonic stages of which are abundant and well-preserved in the slides. Results extend our knowledge of the early fossil record of Fungi, providing compelling evidence of a conserved trophic role for chytrids in one of the earliest well-documented freshwater communities.

## Materials and Methods

We examined historical collections of Rhynie Chert preparations made during the early part of the 20th century primarily to document the fossil plants. We studied a series of thin sections housed at the Natural History Museum London (NHMUK V 15641, NHMUK V 16429, NHMUK V16432, NHMUK V16433, NHMUK V 67866, NHMUK V67867, NHMUK V 67910), the National Museum of Scotland, Edinburgh (NMS G.1925-9-11, NMS G.1925-9-14), and Naturalis Biodiversity Center, Leiden (Netherlands) (RH 585). Zeiss Axioskop and Nikon Eclipse LV100ND compound microscopes were used to examine and photograph specimens with transmitted light. Depth of field was enhanced through z-stack montage.

Confocal Laser Scanning Microscopy (CLSM) is a form of optical microscopy that yields high resolution images of minute objects [26–28]. This method has been used for imaging chytrid sporangia and spores in cellulose acetate peels [27] and plant and fungal tissues in thin section from the Rhynie Chert [8]. Using the latter as a proof of concept, we performed analyses on a larger scale to document the fungal association here described. The presence of Canada Balsam, which was used as a mounting medium, sometimes caused problems of significant background autofluorescence, but we were able to eliminate this by adjusting the setting and time of acquisition of data, and by applying correction parameters. In addition to the opacity of some of the fossil structures, which can inhibit fluorescence, the major difficulties encountered were (1) the generally thicker petrographic thin sections, (2) the fact that the section of chert was not perfectly flat or equally thick across and (3) the variable thickness and quality of the glass coverslips. We were able to work around this problem using 40× and 60× lenses and by zooming on the object. CLSM represents an accurate, non-destructive methodology for collecting 3D morphological data on thin sections of chert.

We acquired confocal images with a Nikon A1-Si laser-scanning confocal microscope. Images were recorded with pixel dimensions of 0.31 μm. Autofluorescence of the samples was excited with the following laser lines: 405-nm line of 100 mW cube laser (Coherent Inc., USA, http://www.coherent.com), 488-nm line of 50 mW sapphire laser (Coherent Inc., USA), 561-nm line of 50 mW sapphire laser (Coherent Inc., USA) and 640-nm line of 40 mW cube laser (Coherent Inc., USA). The quantum efficiency (QE) of the detectors was 20–25%. Autofluorescence signal was collected with 4 photomultiplier-type detectors at the following wavelength emission windows: 425–475 nm for the 405 nm laser, 500–550 nm for the 488 nm laser, 570–620 nm for the 561 nm laser and 675–725 nm for the 640 nm laser. Samples were visualised with a 29.9 μm (1.2 airy units) confocal pinhole and typically 100–400 z-stacks with optical thickness between 200–300 nm each. The fluorescence signal from each z-stack was projected onto a maximum projection image and used to generate a 3D model of the sample with Nikon NIS-Elements software (www.nis-elements.com).

Nomenclature. The electronic version of this article in Portable Document Format (PDF) in a work with an ISSN or ISBN will represent a published work according to the International Code of Nomenclature for algae, fungi, and plants, and hence the new names contained in the electronic publication of a PLOS ONE article are effectively published under that Code from the electronic edition alone, so there is no longer any need to provide printed copies. In addition, new names contained in this work have been submitted to MycoBank from where they will be made available to the Global Names Index. The unique MycoBank number can be resolved and the associated information viewed through any standard web browser by appending the MycoBank number contained in this publication to the prefix http://www.mycobank.org/MB/. The online version of this work is archived and available from the following digital repositories:PubMed Central, LOCKSS, The Natural History Museum repository.

## Results

**Fig 1:**
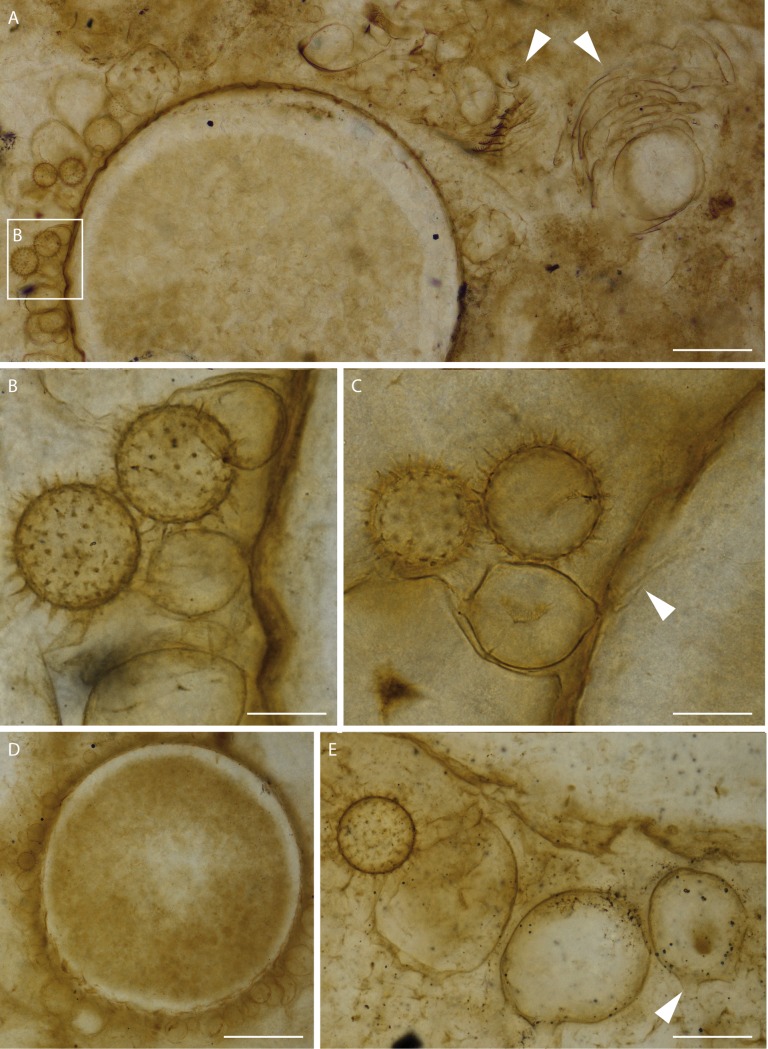
A fungal-animal association from an early freshwater environment (A) The three components in consistent association: wavy-walled rounded substrate structure, fungus and branchiopod resting egg (frame B). Remains of the branchiopod *Lepidocaris rhyniensis* (arrows). (B–C) Resting eggs, zoosporangia and rhizoid penetrating the wavy-walled rounded structure (arrow in C). (B) from the boxed area in (A). (D) Wavy-walled rounded structure filled with the rhizoids. (E) Resting egg and zoosporangia of different sizes bearing discharge papillae (arrow). Scale bars represent 125 μm in (A), 27 μm in (B, C), 175 μm in (D), 37μm in (E). (A, B, C) NHMUK V 16433; (D) NHMUK V16429; (E) NMS G.1925-9-14.

**Fig 2:**
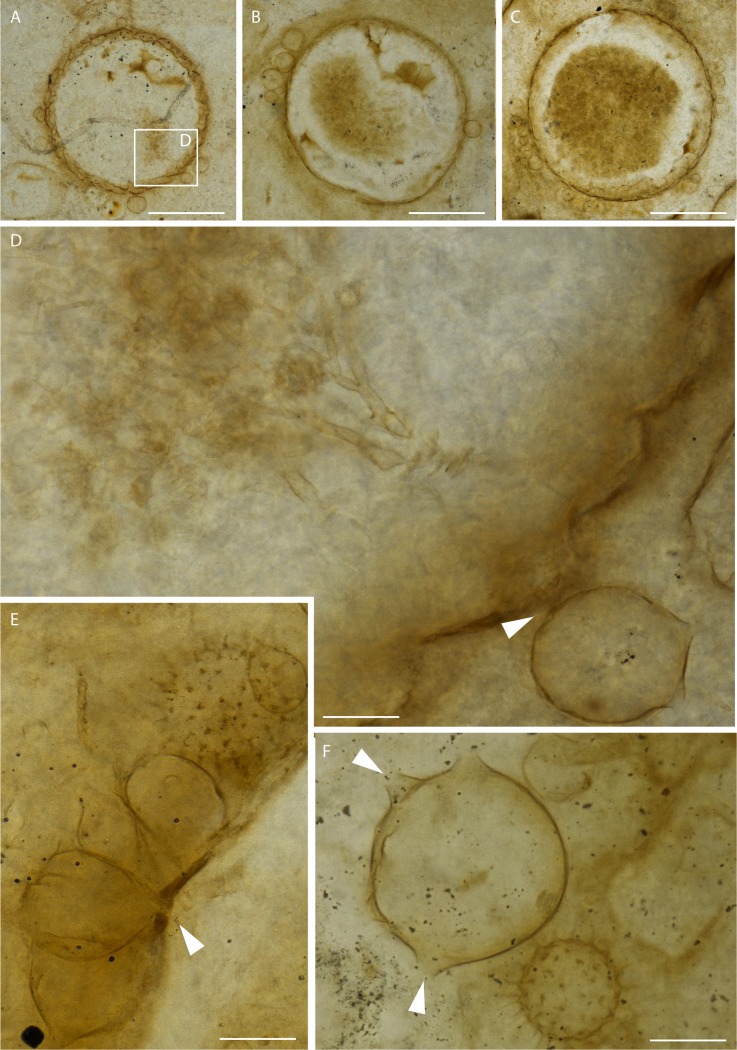
*Cultoraquaticus trewini*: a new Early Devonian Chytridiomycota (A–C) Wavy-walled rounded structures partially or almost entirely filled by the rhizoids. (D) Holotype of *Cultoraquaticus trewini* (NHMUK V 67910). Zoosporangium attached to the wavy-walled rounded structure by a rhizoid (arrow). From boxed area in (A).(E, F) Zoosporangium with attachment (arrow in E), showing discharge papillae (arrows in F).Scale bars represent 340 μm in (A), 195 μm in (B), 290 μm in (C), 23 μm in (D), 21μm in (E), 26 μm in (F).(A, B, D) NHMUK V 67910; (C) NMS G.1925-9-11; (E) NHMUK V16429; (F) NMS G.1925-9-14.

**Fig 3:**
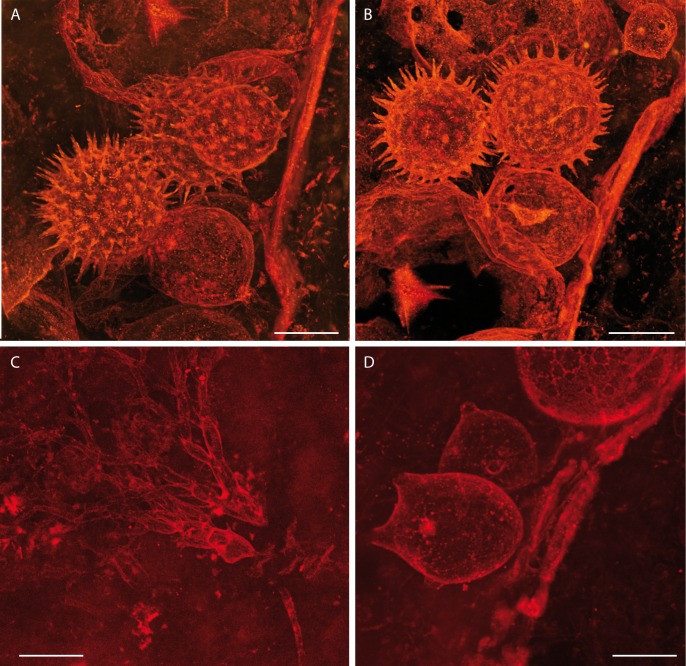
Confocal laser scanning images of resting eggs of *Lepidocaris* and of the fungus *Cultoraquaticus trewini* (A, B) Resting eggs of *Lepidocaris* and fungal zoosporangium showing discharge papillae (See also S1 Movie). (C) Endobiotic and dichotomously branched rhizoids. (D) Zoosporangia showing discharge papillae Scale bars represent 25 μm in (A) and (B), 20 μm in (C) and (D). (A, B) NHMUK V 16433; (C) NHMUK V 67910; (D) NHMUK V 15641.

We studied fossils from thin sections of collections made from Rhynie Chert blocks. Radiometric dating places the age of the Rhynie Chert at 407.6 ± 2.2 My (see discussion in [29] and references cited therein), corresponding to the Early Devonian. We report various associated components of a freshwater environment including remains of the branchiopod crustacean *Lepidocaris rhyniensis* (Fig 1A). The green alga *Palaeonitella* and degraded parts of the plant *Asteroxylon mackiei* are common in these sections but neither occurs in direct association with the organisms reported herein. A new fungus, *Cultoraquaticus trewinii* gen. et sp. nov., a representative of Chytridiomycota, is documented interacting with large, thick-walled rounded structures of unknown affinity. The fungus is represented by zoosporangia that occur outside of these structures (Figs 1, 2, 3A, 3B and 3D) and rhizoidal systems connected to the sporangium that penetrate and fill them (Figs 1C, 2D, and 3C). Associated with the zoosporangia, but without connection to them, are pervasively spinose structures that we interpret as resting eggs of *Lepidocaris rhyniensis* (Figs 1, 2E, 2F and 3A,).

### Systematics of the fungus

The new fungus:

*Cultoraquaticus trewini* Strullu-Derrien 2016 gen.nov. [urn: lsid: Mycobank.org: names: 815878] et sp. nov. [urn: lsid: Mycobank.org: names: 815879].

Kingdom: Fungi

Phylum: Chytridiomycota

Class: Chytridiomycetes

Genus: *Cultoraquaticus* Strullu-Derrien 2016, gen. nov. [urn: lsid: Mycobank.org: names: 815878].

Diagnosis: monocentric, eucarpic thallus; zoosporangia epibiotic with multiple discharge papillae; rhizoids endobiotic and repeatedly branched.

Species: *C*. *trewini* Strullu-Derrien 2016, sp. nov. [urn: lsid: Mycobank.org: names: 815 879].

Diagnosis: as in generic diagnosis. Epibiotic zoosporangium bearing 2–6 apical or subapical papillae; endobiotic rhizoidal system consisting of rhizoids ranging from 6 μm at their base to less than 0.5 μm at tips.

Etymology: genus name refers to the aquatic habitat of the fungus. The specific epithet honours Nigel Trewin for his contribution to the understanding of the geology of the Rhynie Chert.

Holotype *hic designatus*: specimens in slide n° NHM UK V 67910 from the collections at the Natural History Museum, London (Fig 2A and 2D and Fig 3C).

Paratypes: specimens in slides n° NMS G.1925-9-11, NMS G.1925-9-14 from the collections at the National Museum of Scotland, Edinburgh, and NHMUK V 15641, NHMUK V 16429, NHMUK V16432, NHMUK V16433, NHMUK V 67866, NHMUK V67867, RH 585 from the collections at the Natural History Museum, London.

Locality: Rhynie, northwest of Aberdeen (Scotland). Age: Lower Devonian (c. 407 Myr ago [29]).

Mycobank [30, 31] nos: MB 815 878 (genus), MB 815 879 (species).

### Description of the fungal association

Two distinctive elements, interpreted as belonging to the same fungus, are described separately below (types 1 and 2). They are associated with large, rounded to oval substrate structures (hereafter substrate) in 10 thin sections. The type 1 element is external to the substrate whereas type 2 is found inside. Up to 20 substrate structures are present per slide, their diameter varying from 645 to 839 μm (Fig 1A and 1D and Fig 2A–2C) for the rounded forms and from 588 x 694 to 806 x 650 μm for the oval ones; the latter are far fewer. The wall is wavy (Fig 2D), thick, and dark (Fig 2A).

**Type 1** (external). Rounded to sometimes ovoid, smooth walled, bearing 2–6 distal papillae 6–10 μm in length (Fig 1B, 1C and 1E; Fig 2D and 2F and Fig 3A, 3B and 3D). We interpret these external structures as zoosporangia. Their diameters vary from 34 to 90 μm (exceptionally 118 μm) when round and from 39 x 47 to 62 x 79 μm when ovoid; they sometimes appear deformed, as though already empty of zoospores when fossilized. Zoosporangia were attached to the substrate (Fig 1C and 1E; Fig 2D and 2E and Fig 3A and 3B) by a 2.5 μm diameter rhizoid (Fig 1C, Fig 2D and 2E and Fig 3C).

**Type 2** (internal). Inside the wavy-walled substrate is a network of brownish, repeatedly branched filaments (Fig 2A and 2D and Fig 3C) interpreted as a rhizoidal system. The base of the rhizoidal system is narrow (2.5 μm diam.). It then branches and enlarges; there is no septa or pseudosepta below the branching. Individual filaments branch first rather dichotomoustly and taper unevenly from 6 μm to 3 μm to 1.8 μm to less than 0.5 μm diameter at the tip (Fig 2D). Bifurcations in the rhizoids are more frequent towards the distal end and agglomerate to fill the substrate. The latter appear empty of contents other than the rhizoids that partially (Fig 2A and 2B) or almost entirely (Fig 1A and 1D and Fig 2C) fill the space.

### Description of the resting eggs

**Fig 4:**
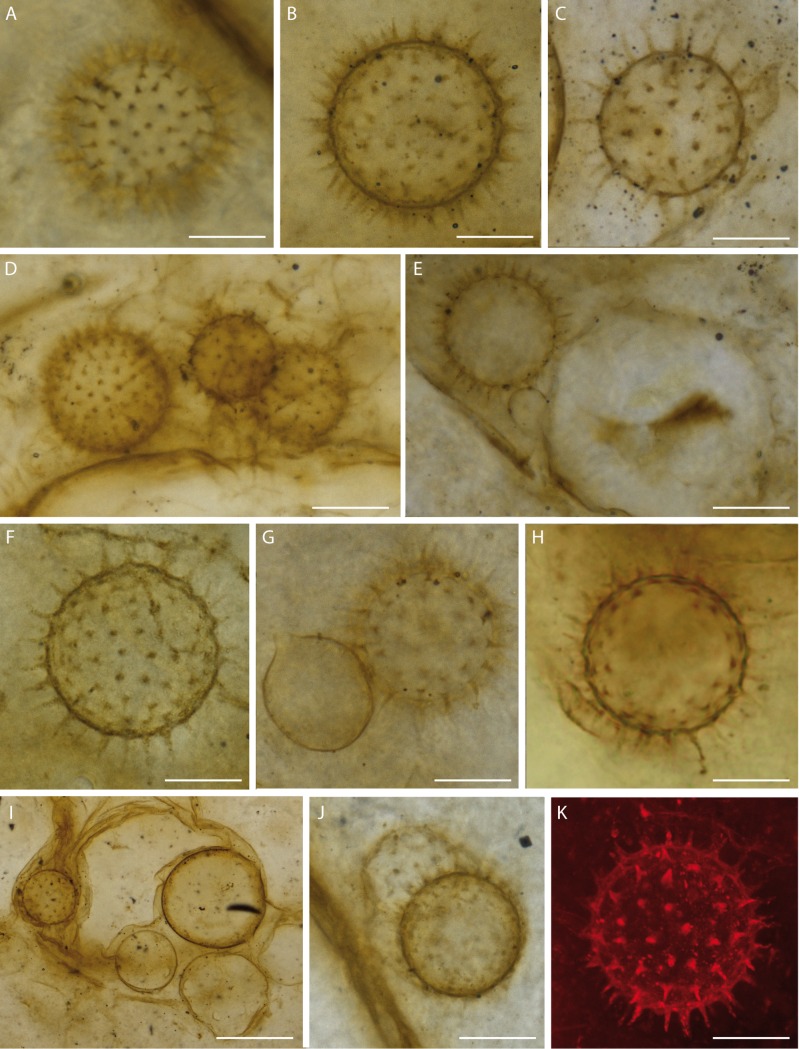
Resting eggs of the branchiopod crustacean *Lepidocaris rhyniensis*. (A–J) Light microscopy, (K) Confocal laser scanning microscopy. Scale bars represent 22 μm in (A), 18 μm in (B, H), 17 μm in (C, F), 30 μm in (D), 27 μm in (E, K), 15 μm in (F), 23 μm in (G), 45 μm in (I), 25 μm in (J). (A, H) NHMUK V 67867; (B) NHMUK V 67910; (C, E) NHMUK V 15641; (D, J) RH 585; (F, G) NHMUK V 16433; (I) NMS G.1925-9-14; (K) NMS G.1925-9-11.

Globular, spiny structures are 42.2–56.3 μm in diameter (including the projections), their regularly spaced, pointed projections are 5.2–7.8 μm long, and 1.8–2.0 μm wide at the base (Fig 1A–1C and 1E, Fig 2E and 2F and Fig 3A and 3B, Fig 4 and S1 Movie). We interpret these as resting eggs of the branchiopod crustacean *Lepidocaris rhyniensis* Scourfield [10] based on their similarity to resting eggs of some extant Branchiopoda and their association with abundant remains of *L*. *rhyniensis*. They regularly occur near, but not attached to, the wavy-walled substrate, mostly distributed among the fungal zoosporangia. Some are packed in groups of three to five, in which case they are not all visible in one focal plane; others occur singly or in groups of two (Fig 1, Fig 3B, Fig 4 and S1 Movie).

## Discussion

### Affinity of the fungus

Chytrids are known from the Rhynie Chert and several chytrid morphotypes and life strategies have been identified, including epibiotic and endobiotic holocarpic and eucarpic forms associated with aquatic algae, land plants, and land plant and fungal spores [8–10, 14–20]. Among the latter are both monocentric and polycentric forms [6, 8]. None of the described monocentric eucarpic species develops an extensive rhizoidal system as observed for *Cultoraquaticus trewini*. *Krispiromyces discoides* [9], associated with the freshwater alga *Palaeonitella cranii*, is somewhat similar to our fungus. Both had eucarpic epibiotic zoosporangia and an endobiotic rhizoidal system and they lived in freshwater environments, but *C*. *trewini* differs from *K*. *discoides* in the shape of the zoosporangia and papillae, the number of papillae per zoosporangium, and the lack of primary and secondary apophyses in its rhizoidal system. *C*. *trewinii* is also similar to *Illmanomyces corniger* [20], both having eucarpic, epibiotic zoosporangia and an endobiotic rhizoidal system. However the number and shape of discharge apparatuses differ between the two species. *I*. *corniger* usually has more than three prominent discharge tubes (30 μm long) whereas *C*. *trewinii* usually has two discharge papillae that seldom emerge from the zoosporangium more than 10 μm. The host substrates and extent of the rhizoidal systems also differ, with *I*. *corniger* having a less developed rhizoidal system in what was identified as a fungal spore, probably glomeromycotan. We consider that these differences justify erecting a new genus and species.

Compared with modern forms, *C*. *trewiniii* shares similarities with members of the Spizellomycetales [32] and the Rhizophydiales [33]. Whereas the former are primarily terrestrial and have rhizoids that measure more than 0.5 μm at their tips, the Rhizophydiales may be either aquatic or terrestrial and have rhizoidal tips less than 0.5 μm at their tips. What was once the morphological genus *Rhizophydium* (e.g. [34]) is now the order Rhizophydiales [35] and extant genera in this order have morphologies that overlap with *C*. *trewinii*. Modern *Rhizophydium* species have a simple thallus composed of a monocentric, epibiotic, spherical and pored sporangium bearing a single rhizoidal axis that branches [36]. These morphological characters can be observed for example in *Rhizophydium zoophthorum*, which grows on rotifer eggs [37] and resembles *C*. *trewinii* except that the latter had multiple discharge papillae. *R*. *hyperparasiticum*, parasitic on a chytrid thallus [36, 37], also shares with *C*. *trewinii* epibiotic zoosporangia with papillae and a richly branched endobiotic rhizoidal system. Species in Rhizophydiales are distributed worldwide and occur as parasites or saprobes of algae, various fungi, spores of ferns, pollen of higher plants, microscopic animals and liver fluke eggs, both in soils and in water [33, 34]. Given its aquatic habitat, narrow rhizoidal tips, and resemblance to some modern members [36, 37], *Cultoraquaticus trewinii* might represent an early member of Rhizophydiales.

### The earliest branchiopod resting eggs

**Fig 5:**
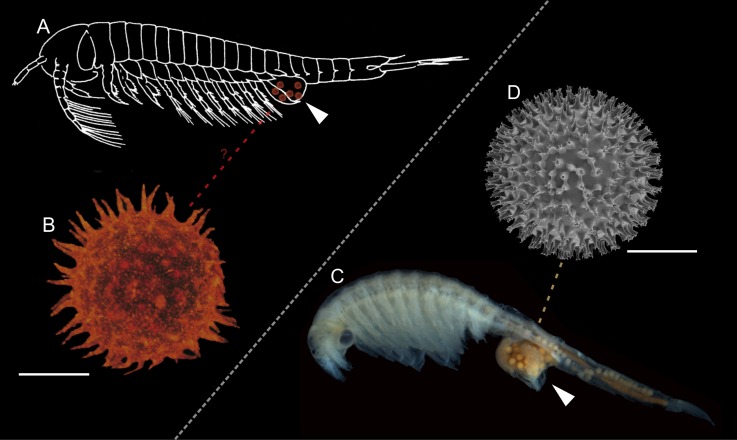
Schematic reconstruction. *Lepidocaris rhyniensis* (A) and its resting egg (B) compared to modern anostracean *Linderiella occidentalis* (C) and resting egg of *Linderiella santarosae* (D). Brood pouch is indicated by an arrow. Scale bars represent 20 μm in (B) and 95 μm in (D).

The globular, spiny structures have some resemblance to resting spores of chytrids e.g. [38] but we dismiss this identity because we found no evidence of a connection to a rhizoidal system or an attachment by a conjugation tube to a donor thallus, which should be present were these chytrid resting spores (see for example figure 162, plate 27 and other examples on plates 25 and 26 in [39]). Rather we interpret these structures free of any attachment as branchiopod resting eggs. Their co-occurrence with abundant remains of *Lepidocaris rhyniensis*, including a series of post-embryonic stages [10, 40, 41], suggests this species as the likely identity. Resting eggs have highly variable morphology in Branchiopoda, but the uniformly spinose morphology in the Rhynie material most closely resembles the egg cysts of various members of Anostraca (fairy shrimp) [42, 43]. However, they also resemble at least one species of Laevicaudata (smooth clam shrimp), *Lynceus simiaefacies* [44]. Their size is siginificantly smaller than egg cysts in modern *Linderiella* (Anostraca) (1/5 of the diameter) (Fig 5B and 5D), which is in accordance with adults of *Lepidocaris rhyniensis* being much smaller (about 3 mm) (Fig 5A) than all modern anostracans (usually 6–25 mm) (Fig 5C). The ontogenetically earliest larva (metanauplius) of *L*. *rhyniensis* reported so far is probably not a hatching stage as it has four somites and is about 0.3 mm in length [10, 40], which is significantly larger than the resting eggs reported here. An alternative candidate for association with the resting eggs are unnamed nauplius larvae described from the Windyfield chert, a slightly younger site near Rhynie [45]. As these larvae are ontogenetically earlier than any known larvae of *Lepidocaris*, they may be a better fit size-wise for the Rhynie resting eggs, but we exclude them since they were shown not to be branchiopod larvae [45] and, unlike *Lepidocaris*, they do not occur in our Rhynie slides.

Several characters uniquely shared by *Lepidocaris* and Anostraca support a position of the Devonian taxon in the anostracan stem-group [46], although an alternative placement in the stem-group of Branchiopoda has also been suggested [47]. The resemblance of the Rhynie Chert spiny structures to anostracan cysts provides a circumstantial argument for an anostracan identity for *Lepidocaris*. Resting eggs have recently been identified in branchiopods from the Upper Devonian (Famennian), including species of Notostraca and Spinicaudata [48]. The former, measuring ca 71 μm, are closer in size to the Rhynie Chert specimens than are extant branchiopod resting eggs. The fossil resting eggs of these orders are clustered in the usual position of brood pouches of extant branchiopods, which is between the limbs (Notostraca) or under the carapace (Spinicaudata). The resting eggs from the Rhynie Chert were not preserved in direct association with brooding structures (e.g., brood pouch) but are better preserved than the Upper Devonian material. Their attribution to *Lepidocaris* indicates that the branchiopod reproductive strategy of encysted eggs dates to at least the Early Devonian and was employed in the earliest well-preserved freshwater ecosystem.

### Evidence of a functional « mycoloop » in early freshwater environments

The Rhynie Chert is a remarkable fossil site both in terms of organismal diversity and the quality of preservation. Although algae have been studied and Fungi have been described as saprotrophs or parasites of charophytes [9], animal interaction with fungi has received less focus, studies being restricted to coprolites [1, 49]. Considering their regular shape and thick wall, the large wavy-walled substrate in our material would have been robust. When they are not colonized by the fungus, they appear empty, suggesting that the original content was soft tissue lost to decay. Their surfaces might have been sticky, explaining why the resting eggs are found close to them. They are spheroidal not tubular. This is clearly demonstrated by the number of perfectly rounded sections of different diameter observed—which would not have been the case were they tubular. The few oval ones observed are slightly dorsoventrally compressed. Their spheroidal form is also evident from image stacks taken at different focal planes. This eliminates the possibility that the substrate was a branch of the alga *Palaeonitella* in cross section. We dispute algal affinity more generally because of the size and lack of wall ornamentation, and plant affinities cannot be substantiated because diagnostic characters such as tracheids are absent. The possibility that the substrate was a fungal spore likewise meets with a lack of diagnostic characters (e.g., wall layers) or attachment structures (e.g., subtending hypha). An identity as animal remains is possible but no characters of particular groups are identified. Despite the uncertain affinity of the substrate, its consistent colonisation by a chytrid fungus provides insights into a trophic interaction. Zoosporangia and rhizoids develop concurrently in most extant chytrids; the rhizoids absorb nutrients, which provide the energy for the sporangia to enlarge. We observed different stages of colonisation of the substrate by the rhizoids (e.g. Fig 2A–2C). When the rhizoids extended inwards to leave empty or weakened spaces, the substrate did not collapse, which suggests that it was still alive at the time of fossilization. On that basis we tend to favour the parasitic hypothesis, the fungus using the content of the substrate to obtain its nutrients, although saprotrophism cannot be excluded.

Based on abundance and constant association with its substrate described above, *Cultoraquaticus trewini* is inferred to have been an important part of the aquatic food web of the Rhynie Chert ecosystem. Branchiopods were another dominant component of this ecosystem, the fossils being frequently disarticulated but without decay induced by fungi or traces of damage caused by other organisms. As recently suggested for the branchiopods from the Upper Devonian [48], temporary pools were likely an environment devoid of predators. In modern aquatic ecosystems parasitic chytrids are the dominant parasites [50]. Chytridiomycota reproduce through the production of motile spores (zoospores). These provide excellent food for zooplankton (e.g. cladoceran crustaceans (*Daphnia*) and copepods) in terms of size, shape, and nutritional quality. They may become particularly important when large, inedible food dominate the community; nutrients within host cells are transferred to zooplankton via the zoospores. This pathway has recently been formally termed the « mycoloop », with saprotrophic as well as parasitic chytrids being implicated [25]. The new chytrid here described occurred in the vicinity of the branchiopod remains. As observed in modern systems, it is therefore probable that *C*. *trewini* played a role in a mycoloop that transferred nutrients obtained from the substrate to *Lepidocaris* in this early freshwater ecosystem. Based on an analysis of the morphology of its feeding appendages, *Lepidocaris* has convincingly been suggested to have scraped material off the substratum on which it fed [51]. Such material would inevitably have contained a mixture of decaying plant, animal, and fungal elements.

## Supporting Information

S1 Movie Resting eggs of *Lepidocaris* and fungal zoosporangium. Movie of Fig 3B; note the discharge papillae of the sporangium.
(AVI)
